# Impact of cerebral collateral flow on stroke outcomes after carotid stenting

**DOI:** 10.1002/acn3.51894

**Published:** 2023-09-01

**Authors:** Liang‐Ju Chen, Chi‐Kuang Liu, Shih‐Chun Wang, Ta‐Tsung Lin, Yang‐Hao Ou, Chih‐Ming Lin

**Affiliations:** ^1^ Department of Health Business Administration Hungkuang University Taichung City Taiwan; ^2^ Department of Medical Imaging Changhua Christian Hospital Changhua City Taiwan; ^3^ Vascular and Genomic Research Center Changhua Christian Hospital Changhua City Taiwan; ^4^ Department of Neurology Changhua Christian Hospital Changhua City Taiwan; ^5^ Department of Mathematics National Changhua University of Education Changhua City Taiwan; ^6^ Graduate Institute of Statistics and Information Science National Changhua University of Education Changhua City Taiwan; ^7^ Department of Post‐Baccalaureate Medicine, College of Medicine National Chung Hsing University Taichung City Taiwan; ^8^ Department of Social Work and Child Welfare Providence University Taichung City Taiwan; ^9^ Department of Nursing, College of Nursing Hungkuang University Taichung City Taiwan

## Abstract

**Objective:**

Internal carotid artery stenosis is a main contributor to recurrent ischemic stroke. This study aimed to evaluate associations between recurrent stroke and changes in prestenting flow direction in the primary collaterals or both primary and secondary collaterals, and the potential interaction between extra‐ and intracranial arteries.

**Methods:**

This longitudinal study recruited stroke patients without intracranial stenosis who underwent right‐side carotid stenting between 2011 and 2019. The main study outcome was recurrent stroke. Predictive factors were anterior circulation flow direction change (ACFDC), posterior circulation flow direction change, and reversal of ophthalmic artery/leptomeningeal anastomosis (ROALA) detected by transcranial color‐coded duplex (TCCD) before carotid stenting. Patient follow‐up was 9 years. Risk factors for recurrent stroke were identified by Kaplan–Meier plot and Cox regression analyses.

**Results:**

A total of 234 patients (mean age 70.88 ± 10.3 years, 86.32% male) were included, and 115 had recurrent stroke. Kaplan–Meier plot showed that patients with left ACFDC and ROALA had worse outcomes than those with ACFDC only, while patients with left ACFDC had worse outcome than those with right ACFDC (both *p* < 0.001). Cox regression analysis showed that recurrent stoke was associated with ACFDC at right (hazard ratio [95% CI]: 20.988 [2.549–172.790], *p* < 0.01), left (151.441 [20.100–1140.993], *p* < 0.001), and both sides (144.889 [19.089–1099.710], *p* < 0.001).

**Interpretation:**

Anterior circulation flow direction change is significantly associated with recurrent stroke in patients with unilateral carotid stenosis. Patients with ACFDC and ROALA together have worse outcomes compared to those with ACFDC only. Prestenting TCCD images help provide definitive information to predict outcomes after carotid stenting.

## Introduction

Carotid stenosis is a major contributor to ischemic stroke events.^1^, ^2^ Prompt management, including carotid stenting or surgical carotid endarterectomy, is the main options employed to prevent recurrent stroke. However, long‐term outcomes or recurrent stroke episodes after the first‐time stroke event after carotid stenting treatment have not been fully investigated.

Our previous studies reported that the detection of the reversal of the ophthalmic artery via extracranial carotid duplex examination predicted unfavorable outcomes after stenting treatment based on results of the modified Rankin Scale (mRS) at three months after the treatment.^3^, ^4^, ^5^ Another study also revealed the coexistence of intracranial large artery stenosis, specifically middle cerebral artery stenosis (>50% lumen reduction) identified using magnetic resonance imaging and arteriography (MRI/ MRA) with weak functional outcomes assessed by both Barthel Index and mRS.^6^, ^7^ Transcranial color‐coded duplex (TCCD) is a noninvasive clinical neuroimaging modality with the advantages of mobility, repeatability, and noncontrast modality. Although its clinical use in combination with extracranial carotid duplex examination can thoroughly assess stroke patients' intracranial and extracranial basal artery vasculature conditions, it remains an underused and neglected tool. Through the utilization of TCCD and carotid duplex examination, first‐line clinicians are able to detect the activation of primary and secondary collaterals in real time and subsequently design an appropriate treatment program. In this longitudinal study, our team collected the long‐term data of stroke patients with carotid stenosis who received carotid stenting treatment. During hospitalization, MRI and MRA were arranged to pinpoint the exact stroke location and identify major intracranial stenosis or tandem vasculature stenosis. Patients were followed up to 9 years after the first‐time stroke episode.

The primary aim of this study was to gauge the significance of cerebral collateral activation associated with stroke recurrence in patients who underwent unilateral carotid stenting. Second, we attempted to compare the impact of the insufficiency of primary collaterals, or both primary and secondary collaterals, in order to predict recurrence risk and the interval between the first and recurrent stroke episodes.

## Methods

### Patient selection

**Figure 1:**
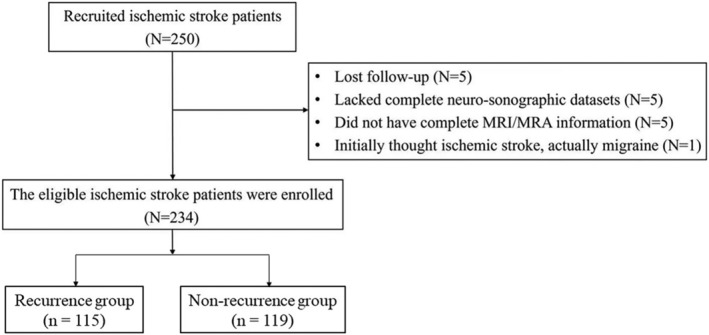
Flowchart of patient selection.

A total of 234 consecutive patients from the outpatient clinics and emergency departments of Changhua Christian Hospital or transferred from a branch hospital and who were scheduled to undergo right‐side carotid stenting between 2011 and 2019 were recruited. Inclusion criteria were as follows: (1) age ≥ 18 years; (2) first‐time ischemic strokes; (3) with evidence of >70% carotid stenosis via computed tomography angiography/perfusion (CTA/P) and carotid duplex examinations; (4) with no major intracranial stenosis documented by magnetic resonance imaging/angiography (MRI/A); (5) no etiology of other clinical illness or cardiogenic‐induced etiologies; and (6) without intravenous thrombolytic therapy or intra‐arterial thrombectomy treatments (intravenous r‐tPA and IA procedures). Exclusion criteria were as follows: (1) patients with cerebral hemorrhage, cerebral arteriovenous malformations, aneurysms, or bilateral moderate–severe carotid stenosis; (2) follow‐up less than 12 months. The enrolled patients were hospitalized for medical treatments along with baseline clinical laboratory (hematology/biochemistry) work‐ups. Ischemic stroke was confirmed by the diffusion‐weighted sequence of MRI. Diagnostic digital subtraction angiography (DSA) was arranged during hospitalization to gauge the degree of carotid stenosis. Patients were stented within 1 month after the index episode (initial stroke event) (Fig. 1).

Before carotid stenting, carotid stenosis was evaluated using CTA/P and carotid duplex examination. Transcranial color‐coded duplex was arranged when any anterior circulation changes or posterior flow changes were noted. MRI/MRA was performed to confirm the localization of stroke, while MRA was also performed to confirm that no major basal arteries had stenosis >50%. Baseline clinical laboratory testing (hematology/ biochemistry) was performed and neuroradiological examinations were carried out for patients in both the emergency room and neurological ward. Patients' comorbidities, neurological and physical examinations, relevant drug history, and personal medical history were recorded during hospitalization.

### Ethics statement

The protocol of this study was reviewed and approved by the Institutional Review Board of Changhua Christian Hospital (CCH IRB No.: 211210). All included patients were waivered of the signed informed consent due to the retrospective study in nature.

### Outcome measurements

The primary outcome was the occurrence of recurrent stroke during the 9‐year follow‐up after right carotid stenting.

### Carotid duplex

A Philips iE33 7‐Mhz linear transducer (Philips Inc., Amsterdam, Netherlands) was used to examine the cervical carotid artery as previously described.^8^ The transducer was placed on patients' necks as they were asked to slightly tilt their heads contralaterally. Cross‐sectional B‐mode scanning and longitudinal screening were performed to identify and confirm intraluminal plaques and degree of stenosis, respectively. According to the International Classification System, plaque was classified into subtypes 1, 2, 3, or 4.^9^ The recorded parameters included peak systolic velocity (PSV), end diastolic velocity (EDV), and resistance index (RI) (PSV—EDV/PSV) of the bilateral common carotid artery, internal carotid artery (ICA), external carotid artery (ECA), and ophthalmic artery as well as reversal of ophthalmic artery flow. Forward flow was defined as blood flow directed away from the stenotic ipsilateral carotid artery and reverse flow was defined as blood flow into the carotid artery.^4^, ^5^ The level of carotid stenosis was evaluated based on the method used in the European Carotid Surgery Trial.^10^, ^11^, ^12^

### Computed tomography angiography/perfusion scan (CTA/P imaging)

A second‐generation dual‐source CT scanner (SOMATOM Definition Flash, Siemens Healthcare, Forchheim, Germany) was used to perform CTA examinations. Perfusion datasets were then processed using a Siemens Multimodality Workplace Workstation (Siemens Medical, Germany), which calculated mean transit time (MTT), cerebral blood volume (CBV), cerebral blood flow (CBF), and time to peak (TTP). Curves of arterial input and venous outflow were both analyzed to ensure dataset completeness. The CTP parameters were defined as follows: (1) dMTT: ipsilateral MTT − contralateral MTT. (2) MTT ratio: ipsilateral MTT/contralateral MTT. (3) MTT index: (ipsilateral MTT − contralateral MTT)/ contralateral MTT. (4) dCBV: ipsilateral CBV − contralateral CBV. (5) CBV ratio: ipsilateral CBV/contralateral CBV. (6) CBV index: (ipsilateral CBV − contralateral CBV)/ contralateral CBV. (7) dCBF: ipsilateral CBF − contralateral CBF. (8) CBF ratio: ipsilateral CBF/contralateral CBF. (9) CBF index: (ipsilateral CBF − contralateral CBF)/contralateral CBF. (10) dTTP: ipsilateral TTP − contralateral TTP. (11) TTP ratio: ipsilateral TTP/contralateral TTP. (12) TTP index: (ipsilateral TTP − contralateral TTP)/contralateral TTP.

### Magnetic resonance imaging/angiography (MRI/A)

A 3 T‐ (MagnetomVerio, Siemens Healthcare, USA) or 1.5‐T imager (MagnetomAera, Siemens Healthcare) with a cervical coil was used to perform MRI/A examination.

### Transcranial color‐coded duplex (TCCD)

The cerebral basal arteries were examined using a Philips iE33 2‐Mhz transducer (Phillips Healthcare, Amsterdam, the Netherlands) placed above zygomatic arch while patients were in a supine position. The protocol of our hospital is to assess the basal arteries from both right and left side of insonatic windows. The temporal insonation of both sides allows the assessment of terminal internal carotid artery, middle cerebral artery, anterior cerebral artery, anterior and posterior communicating arteries, and posterior cerebral arteries. The flow direction, systolic and end diastolic velocity, mean flow velocity, and pulsatilty index values were recorded automatically. The transorbital insonation was arranged only when flow direction changes in terminal ICA and/or ophthalmic artery were not certain via carotid duplex. The transforaminal insonation was performed to assess flow direction and velocity difference of intracranial basilar artery and terminal intracranial portion of bilateral vertebral arteries. Anterior circulation flow direction change (ACFDC) was defined as TCCD‐detected flow direction changes of either the anterior cerebral artery, anterior communicating artery, distal part of internal carotid artery, or proximal part of middle cerebral artery. Posterior circulation flow direction change (PCFDC) was defined as TCCD‐detected flow direction changes of either the posterior cerebral artery, posterior communicating artery, or distal part of basilar artery or intracranial vertebral artery.

### Statistical analysis

All statistical analyses were performed using the statistical package SPSS for Windows (Version 21.0, SPSS Inc., IBM Corp., Armonk, NY, USA), and the results were considered statistically significant at *p* < 0.05. Descriptive data on patients' characteristics are expressed as numbers (n) and percentages (%). Chi‐squared test was used to compare patients' characteristics between recurrent stroke and nonrecurrent stroke groups. Chi‐squared test was also used to examine the significance of differences in intracranial and extracranial flow conditions. The months of recurrent stroke with intracranial and extracranial flow conditions were compared using one‐way ANOVA.

The Kaplan–Meier method was performed to estimate the probability of recurrence‐free stroke at given times and to clarify patients' outcomes with different intracranial and extracranial flow conditions. The log‐rank test was used to compare the significance of between‐group differences. The Cox proportional hazard model was constructed to evaluate associations between potential risk factors and the odds of recurrent stroke, by estimating hazard ratios (HR) with 95% CI.

## Results

Table 1 shows the baseline demographic and clinical characteristics of the included 234 patients with right‐side carotid stenosis stroke, of whom 115 patients had recurrent stroke episodes. Patients' mean age was 70.88 ± 10.3 years, and 86% were older than 60 years. The rate of recurrent stroke tended to increase with age, and exceeded 50% in patients over 75 years. Most included cases were men (86.32%), but women had a higher rate of recurrent stroke than men (56.25% vs. 48.02%). No significant differences were found in education levels, smoking, BMI, or comorbidities between recurrence and nonrecurrence groups (all p > 0.1).

**Table 1:** Demographic and clinical characteristics and recurrent stroke rate of study population.

Characteristics/variables	*N*	%^1^	Recurrent stroke		*p*
			*N*	%^2^	
Total	234	100.00	115	49.15	0.161
Age (mean, SD)	(70.88, 10.30)				
<60	32	13.68	12	37.50	
61–64	31	13.25	14	45.16	
65–69	31	13.25	13	41.94	
70–74	35	14.96	15	42.86	
75–79	55	23.50	35	63.64	
>80	50	21.37	26	52.00	
Sex					
Female	32	13.68	18	56.25	
Male	202	86.32	97	48.02	
Education					0.651
<6 years	17	7.26	10	58.82	
6 years	72	30.77	37	51.39	
9 years	36	15.38	19	52.78	
12 years	97	41.45	45	46.39	
>12 years	12	5.13	4	33.33	
Smoking					0.625
No	182	77.78	91	50.00	
Yes	52	22.22	24	46.15	
BMI (mean, SD)	(23.70, 3.48)				0.809
Underweight	13	5.56	5	38.46	
Healthy weight	124	52.99	61	49.19	
Overweight	60	25.64	29	48.33	
Obesity	37	15.81	20	54.05	
Comorbidities					
Hypertension	168	71.79	87	51.79	0.197
DM	84	35.90	36	42.86	0.150
CVA	67	28.63	34	50.75	0.756
CAD	39	16.67	21	53.85	0.520
AF	6	2.56	4	66.67	0.440
Liver disease	18	7.69	12	66.67	0.122
CKD	43	18.38	22	51.16	0.770
Gout	22	9.40	12	54.55	0.595

AF, atrial fibrillation; BMI, body mass index; CAD, coronary artery disease; CKD, chronic kidney disease; CVA, cerebrovascular accident; DM, diabetes mellitus; SD, standard deviation.

^1^ Percentage of total patients.

^2^ Percentage of each characteristic/variable with recurrent stroke.

Table 2 demonstrates the correlations between recurrent stroke and flow direction changes in primary and secondary collaterals before carotid stenting. For primary collaterals, the recurrence group had significantly higher proportions of ACFDC at the right side (R‐ACFDC, 62.22% vs. 37.78%, *p* = 0.002) and ACFDC at the left side (L‐ACFDC, 96.36% vs. 3.64%, *p* < 0.001) compared with the nonrecurrence group, but no differences were found in PCFDC (50% vs. 50%, *p* = 0.799). For secondary collaterals, the recurrence group also had a significantly higher proportion of reverse ophthalmic artery/leptomeningeal anastomosis at right side (R‐ROALA, 80% vs. 20%, *p* < 0.001) compared with that in the nonrecurrence group. When combining primary and secondary collaterals for data analysis, it was clearly elucidated that the recurrent stroke group had a significantly higher proportion of ACFDC+R‐ROALA compared with the ACFDC‐only group and nonrecurrent stroke group (93.75% vs. 54.17% and 6.25%, *p* < 0.001).

**Table 2:** Comparison of primary and secondary collaterals between nonrecurrent stroke and recurrent stroke groups.

	*N*	Nonrecurrent stroke		Recurrent stroke		*p*
		*n*	%	*n*	%	
Total	234	119	50.85	115	49.15	
Primary collaterals						
R‐ACFDC						
No	144	85	59.03	59	40.97	0.002**
Yes	90	34	37.78	56	62.22	
L‐ACFDC						
No	124	115	92.74	9	7.26	0.000***
Yes	110	4	3.64	106	96.36	
PCFDC						
No	120	62	51.67	58	48.33	0.799
Yes	114	57	50.00	57	50.00	
Secondary collaterals						
R‐ROALA						
No	139	100	71.94	39	28.06	0.000***
Yes	95	19	20.00	76	80.00	
Re‐grouping collaterals						
Non ACFDC	82	81	98.78	1	1.22	0.000***
Only ACFDC	72	33	45.83	39	54.17	
ACFDC + R‐ROALA	80	5	6.25	75	93.75	

ACFDC, anterior circulation flow direction change; L, left side; PCFDC, posterior circulation flow direction change; R, right side; ROALA, reversal of ophthalmic artery/leptomeningeal anastomosis.

**p < 0.01; ***p < 0.001 using chi‐squared test.

**Figure 2:**
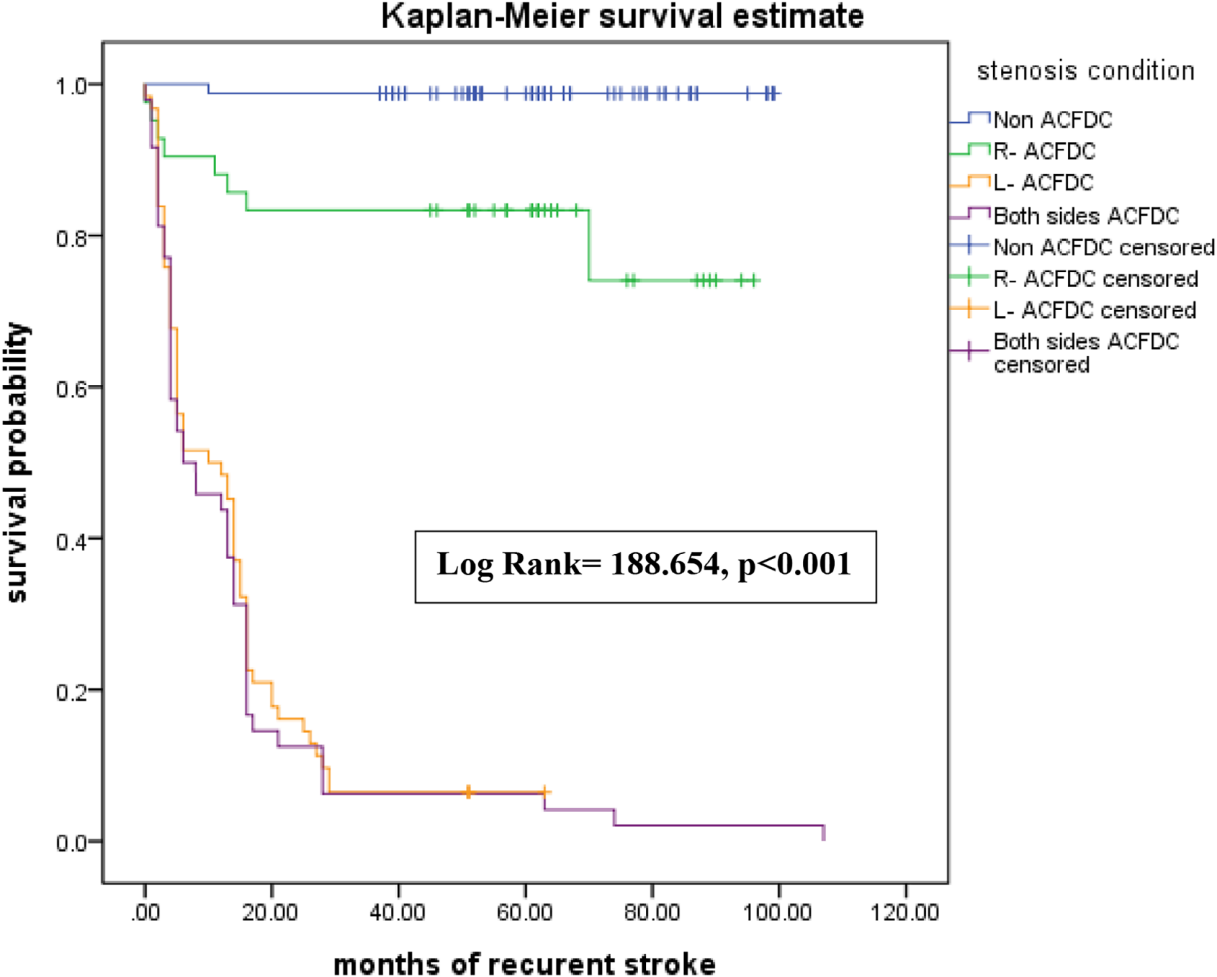
Kaplan–Meier plot of recurrent stroke occurrence for patients with ACFDC within a 9‐year follow‐up from the initial stroke onset.

**Figure 3:**
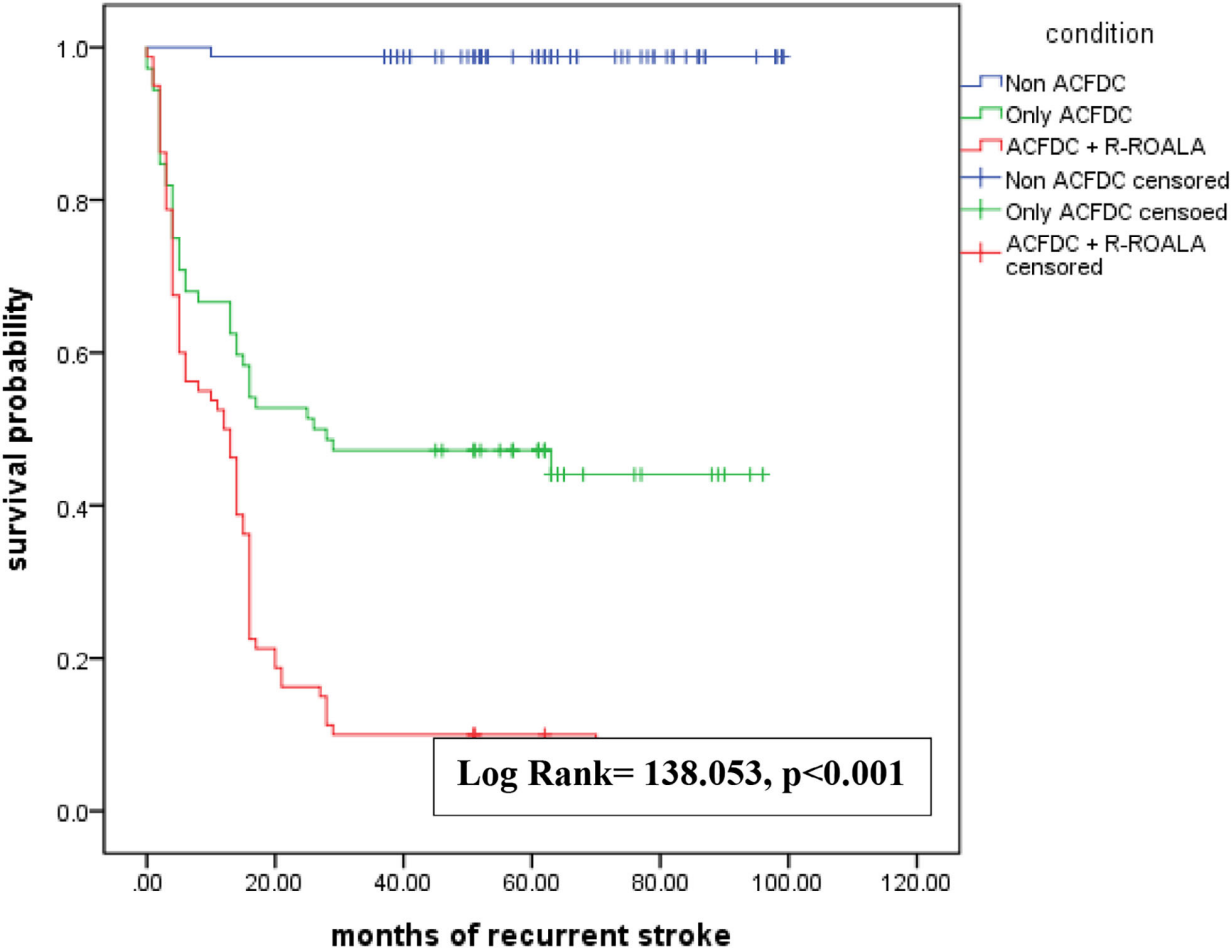
Kaplan–Meier plot of recurrent stroke occurrence for patients with non‐ACFDC, ACFDC, and ACFDC with R‐ROALA within a 9‐year follow‐up from the initial stroke onset.

The associations between intracranial flow change, recurrent stroke, and extracranial flow change are summarized in Table 3. Among patients with recurrent stroke, 50.43% had L‐ACFDC and 41.74% had both‐sides ACFDC (*p* < 0.001). Among patients with R‐ROALA, 42.11% had L‐ACFDC and 32.63% had both‐sides ACFDC (*p* < 0.001). Figure 2 is the Kaplan–Meier plot of recurrent stroke for the four ACFDC groups within 9 years of follow‐up. The L‐ACFDC and both‐sides ACFDC groups had the poorest outcomes, followed by the R‐ACFDC group. The non‐ACFDC group had most favorable outcomes (*p* < 0.001 by log rank test). Figure 3 is the Kaplan–Meier plot of recurrent stroke for activation of primary and secondary collaterals. The ACFDC + R‐ROALA group had the weakest outcomes, followed by the ACFDC‐only group. The non‐ACFDC group had the best outcomes (*p* < 0.001 by log rank test).

**Table 3:** Associations between recurrent stroke, PCFDC, R‐ROALA and ACFDC, stratified by side of ACFDC.

	*N*	Non‐ACFDC		R‐ ACFDC		L‐ACFDC		Both‐sides ACFDC		*χ* ^2^	*p*
		*n*	%	*n*	%	*n*	%	*n*	%		
Total	234	82	35.04	42	17.95	62	26.50	48	20.51		
Recurrent stroke											
No	119	81	68.07	34	28.57	4	3.36	0	0.00	189.163	0.000***
Yes	115	1	0.87	8	6.96	58	50.43	48	41.74		
PCFDC										4.789	0.188
No	120	44	36.67	20	16.67	37	30.83	19	15.83		
Yes	114	38	33.33	22	19.30	25	21.93	29	25.44		
R‐ ROALA										49.475	0.000***
No	139	67	48.20	33	23.74	22	15.83	17	12.23		
Yes	95	15	15.79	9	9.47	40	42.11	31	32.63		

ACFDC, anterior circulation flow direction change; L, left side; PCFDC, posterior circulation flow direction change; R, right side; ROALA, reversal of ophthalmic artery/leptomeningeal anastomosis.

*** *p* < 0.001 using chi‐squared test.

Table 4 presents multivariable Cox regression analyses of recurrent stroke after right carotid stenting. Recurrent stroke was significantly associated with right‐side ACFDC (adjusted HR [aHR]: 20.988, 95% CI: 2.549–172.790, *p* < 0.01), left side (aHR: 151.441, 95% CI: 20.100–1140.993, *p* < 0.001), and both sides (aHR: 144.889, 95% CI: 19.089–1099.710, *p* < 0.001). No statically significant difference was observed in other prestenting parameters. Table 5 shows the time interval in months between the first stroke to the recurrent stroke episode after right carotid stenting. The intervals were 65.20, 35.15, and 16.16 months in the non‐ACFDC, ACFDC‐only, and ACFDC+R‐ROALA groups, respectively (*p* < 0.001).

**Table 4:** Multivariable Cox regression analyses for recurrent stroke.

Risk factor (reference)	Adjusted HR	95% CI	*p*
Sex	0.630	0.340–1.169	0.143
Age	1.001	0.980–1.022	0.908
Education (≤9 years)	0.851	0.547–1.323	0.474
Numbers of disease	0.922	0.766–1.110	0.391
BMI (underweight)			
Healthy weight	1.437	0.295–7.007	0.654
Overweight	0.927	0.189–4.554	0.925
Obesity	1.626	0.318–8.317	0.559
ACFDC			
R‐side	20.988**	2.549–172.790	0.005
L‐side	151.441***	20.100–1140.993	0.000
Both‐sides	144.889***	19.089–1099.710	0.000
PCFDC (No)	0.898	0.561–1.435	0.652
R‐ROALA (No)	1.177	0.721–1.923	0.514
R‐IMT (No)	1.441	0.378–5.496	0.593
L‐IMT (No)	0.995	0.957–1.035	0.811
R‐Plaque index (Cont.)	1.030	0.944–1.124	0.508
L‐Plaque index (Cont.)	0.979	0.908–1.055	0.577

ACFDC, anterior circulation flow direction change; CI, confidence interval; Cont., continuous variable; HR, hazard ratio; IMT, intima‐media thickness; L, left side; PCFDC, posterior circulation flow direction change; R, right side; ROALA, reversal of ophthalmic artery/leptomeningeal anastomosis.

** *p* < 0.01; *** *p* < 0.001.

**Table 5:** Interval from initial stroke onset to recurrent stroke (months).

Variable	Months of recurrent stroke		*F*	*p*	Post hoc test
	Mean	SD			
Total	39.19	31.18	89.766	0.000***	1 > 2 > 3
1. Non‐ACFDC	65.20	19.55			
2. ACFDC‐only	35.15	30.13			
3. ACFDC + R‐ROALA	16.16	20.14			

*p* value determined using one‐way ANOVA of associations. Scheffe method was used for post hoc test.

ACFDC, anterior circulation flow direction change; R, right side; ROALA, reversal of ophthalmic artery/leptomeningeal anastomosis; SD, standard deviation.

## Discussion

The present study showed that prestenting ACFDC is associated with recurrent stroke after right carotid stenting. Existence of insufficient primary and secondary collaterals results in higher recurrent stroke rates compared with insufficient primary collateral only. This is exemplified by the poorer outcomes shown by Kaplan–Meier plot in patients with ACFDC and ROALA than in those with ACFDC only.

When the ICA has severe stenosis, the primary collateral is the first initiated intracranial flow to supply the hypoperfused cerebral hemisphere, and the secondary collateral flow is initiated consequently when the primary collateral in the Willis circle is insufficient.^12^, ^13^, ^14^, ^15^ In the present study, when the right carotid artery had stenosis >70%, the right anterior, middle, and posterior cerebral arteries were first initiated to support the hypoperfused right cerebral hemisphere. Flow direction change of a certain specific artery implies insufficient supply via the primary collateral circulation of the Willis circle. Under the condition of no intracranial stenosis, the abundant branches in the posterior circulation can afford to support the insufficient local flow reflected by PCFDC. However, Connolly et al. reported that the anterior communicating artery is more frequently activated than the posterior communicating artery in patients with unilateral ICA occlusion, suggesting that sufficient flow within the anterior circulation is important for hemodynamic compensation of ICA occlusion.^16^ This is consistent with results of the present study showing that recurrent stroke is associated with ACFDC at either side, but not associated with PCDFC. Flow direction changes in both ACFDC and the right ophthalmic artery imply that primary and secondary collaterals have been recruited to support the flow; therefore, those patients have poorer outcomes than patients with ACFDC only.

The present study also showed that among patients with right carotid stenosis, those with left‐side ACFDC and both sides‐ACFDC have comparable poor outcomes, while those with R‐ACFDC have significantly better outcomes compared to those with L‐ACFDC. Rutgers et al. found that among patients with symptomatic ICA occlusion, those with recurrent stroke had significantly higher total flow to the brain and higher contralateral ICA flow compared to those without recurrent stroke,^17^ which may help to explain our results from patients with severe right ICA stenosis. In patients with R‐ACFDC, increased total flow to the brain and increased left ICA flow both help to support the right cerebral hemisphere; for patients with L‐ACFDC, the final flow to the right cerebral hemisphere is reduced because the increased left ICA flow and flow to the brain are attenuated by insufficient left anterior circulation, which is similar to that in patients with ACFDC at both sides.

Studies have reported associations between ROALA and poorer outcomes in patients with carotid stenosis.^4^, ^5^ The present study showed that ipsilateral ROALA alone was not associated with recurrent stroke. Reversal of ophthalmic artery/leptomeningeal anastomosis is associated with recurrent stroke only with the coexistence of primary collateral insufficiency, because both primary and secondary collaterals have been activated to support the hypoperfused cerebral hemisphere.^6^

The disadvantages of transcranial color‐coded duplex sonography (TCCS) include that it is operator‐dependent, time‐consuming, and requires advanced training in the medical institution to accurately perform the examination. However, TCCS allows noninvasive assessment of intracranial collaterals. The present study used TCCS and carotid duplex to study the impact of intra‐ and extracranial flows as well as primary and secondary collaterals on predicting recurrent stroke.^18^ Flow direction changes of the primary and secondary collaterals as presented by TCCS and carotid duplex provided definitive information by which to predict patients' prognosis after carotid stenting.

### Limitations

The present study has several limitations. First, it was a single‐center study and the recruited patients were all of Asian origin. The results of this project could therefore be applied only to the limited ethnicity and could not be generalized to other populations. Second, antiplatelet drug administration and compliance during the study period was not fully recorded. It remains unknown whether these factors may potentially confound long‐term outcomes. Third, the datasets of biochemistry testing, neuroimaging, and neurosonological tests were recorded at the prestenting period. The datasets from follow‐up were not investigated. Lastly, the current study only recruited patients with carotid stenting procedures instead of endarterectomy, the long‐term effect of endarterectomy remains unknown and yet to be studied.

## Conclusions

Anterior circulation flow direction change before carotid stenting is significantly associated with recurrent ischemic stroke. Patients with ACFDC and R‐ROALA have poorer outcomes compared to those with ACFDC only. Prestenting TCCD images help provide definitive information predicting patients' outcomes after carotid stenting.

## Author Contributions

**Liang-Ju Chen:** Biostatistical analysis, study supervision and manuscript writing; **Chi-Kuang Liu:** Data validation; **Shih-Chun Wang:** Data collection; **Ta-Tsung Lin**: Data collection; **Yang-Hao Ou:** IRB preparation; **Chih-Ming Lin:** Study design, biostatistical analysis, data collection, IRB preparation, and manuscript writing.

## Conflict of Interest

The authors declare that we have no competing interests with any organizations/institutions.
